# Identifying response and predictive biomarkers for Transcranial magnetic stimulation outcomes: protocol and rationale for a mechanistic study of functional neuroimaging and behavioral biomarkers in veterans with Pharmacoresistant depression

**DOI:** 10.1186/s12888-020-03030-z

**Published:** 2021-01-13

**Authors:** Leanne M. Williams, John T. Coman, Patrick C. Stetz, Nicole C. Walker, F. Andrew Kozel, Mark S. George, Jong Yoon, Laura M. Hack, Michelle R. Madore, Kelvin O. Lim, Noah S. Philip, Paul E. Holtzheimer

**Affiliations:** 1 Department of Psychiatry and Behavioral Sciences, Stanford University School of Medicine, 401 Quarry Road, Stanford, CA 94304 USA; 2 Mental Illness Research, Education and Clinical Center, VA Palo Alto Health Care System, 3801 Miranda Ave, Palo Alto, CA 94304 USA; 3 Department of Behavioral Sciences and Social Medicine, Florida State University, 1115 W Call St, Tallahassee, FL 32304 USA; 4 Department of Psychiatry and Behavioral Neurosciences, University of South Florida, 3515 E Fletcher Ave, Tampa, FL 33613 USA; 5 Department of Psychiatry and Behavioral Sciences, Medical University of South Carolina, 96 Jonathan Lucas St. Ste. 601, MSC 617, Charleston, SC 29425 USA; 6 Ralph H. Johnson VA Medical Center, Charleston, SC USA; 7 Department of Psychiatry and Behavioral Sciences, University of Minnesota Medical School, 420 Delaware St SE, Minneapolis, MN 55455 USA; 8 Minneapolis VA Health Care System, 1 Veterans Dr, Minneapolis, MN 55417 USA; 9 Department of Psychiatry and Human Behavior, Alpert Medical School of Brown University, 345 Blackstone Boulevard, Providence, RI 02908 USA; 10 VA RR&D Center for Neurorestoration and Neurotechnology, Providence VA Medical Center, 830 Chalkstone Ave, Providence, RI 02908 USA; 11 Departments of Psychiatry and Surgery, Geisel School of Medicine at Dartmouth, Dartmouth Hitchcock Medical Center, 1 Medical Center Dr, Lebanon, NH 03756 USA; 12 Executive Division, National Center for PTSD, White River Junction VA Medical Center, 215 North Main St., White River Junction, VT 05009 USA

**Keywords:** Major depressive disorder (MDD), Treatment resistant depression (TRD), Repetitive Transcranial magnetic stimulation (TMS), Dorsolateral prefrontal cortex (DLPFC), Functional magnetic resonance imaging (fMRI), Neuroimaging, Biomarker, Cognitive control network, Default mode network (DMN), Veterans

## Abstract

**Background:**

Although repetitive transcranial magnetic stimulation (‘TMS’) is becoming a gold standard treatment for pharmacoresistant depression, we lack neural target biomarkers for identifying who is most likely to respond to TMS and why. To address this gap in knowledge we evaluate neural targets defined by activation and functional connectivity of the dorsolateral prefrontal cortex-anchored cognitive control circuit, regions of the default mode network and attention circuit, and interactions with the subgenual anterior cingulate. We evaluate whether these targets and interactions between them change in a dose-dependent manner, whether changes in these neural targets correspond to changes in cognitive behavioral performance, and whether baseline and early change in neural target and cognitive behavioral performance predict subsequent symptom severity, suicidality, and quality of life outcomes. This study is designed as a pragmatic, mechanistic trial partnering with the National Clinical TMS Program of the Veteran’s Health Administration.

**Methods:**

Target enrollment consists of 100 veterans with pharmacoresistant Major Depressive Disorder (MDD). All veterans will receive a clinical course of TMS and will be assessed at ‘baseline’ pre-TMS commencement, ‘first week’ after initiation of TMS (targeting five sessions) and ‘post-treatment’ at the completion of TMS (targeting 30 sessions). Veterans will be assessed using functional magnetic resonance imaging (fMRI), a cognitive behavioral performance battery, and established questionnaires. Multivariate linear mixed models will be used to assess whether neural targets change with TMS as a function of dose (Aim 1), whether extent and change of neural target relates to and predicts extent of behavioral performance (Aim 3), and whether extent of neural target change predicts improvement in symptom severity, suicidality, and quality of life (Aim 3). For all three aims, we will also assess the contribution of baseline moderators such as biological sex and age.

**Discussion:**

To our knowledge, our study will be the first pragmatic, mechanistic observational trial to use fMRI imaging and cognitive-behavioral performance as biomarkers of TMS treatment response in pharmacoresistant MDD. The results of this trial will allow providers to select suitable candidates for TMS treatment and better predict treatment response by assessing circuit connectivity and cognitive-behavioral performance at baseline and during early treatment.

**Trial registration:**

ClinicalTrials.gov NCT04663481, December 5th, 2020, retrospectively registered. The first veteran was enrolled October 30th, 2020.

## Background

Major depressive disorder (MDD) is the leading cause of disability worldwide [1]. Due to a lack of mechanistically anchored quantitative tests for identifying the correct intervention for individual patients at their first presentation, treatment choice is often a years-long trial-and-error process. One reason for the unpredictability of therapeutic response is the heterogeneity of MDD, both clinically and in terms of underlying neurobiology [2]. While definitions vary, pharmacoresistant or treatment-resistant depression is defined as lack of response to at least one antidepressant trial of adequate dose and duration [3]; up to 50% of MDD patients meet these criteria [4]. Residual depressive symptoms are associated with a higher risk of recurrence, worse functioning, and increased personal and economic burden [5]. Furthermore, pharmacoresistant depression can be life threatening: 30% of patients have one or more lifetime suicide attempts, which is at least twice the rate of those with non-resistant depression [6].

Neural circuits (hereafter circuits) consist of vast numbers of interconnected neurons comprising the anatomical and functional networks of the brain [7]. Circuits involved in cognitive control are promising targets for pharmacoresistant depression. Dysfunctions in the cognitive control network and reciprocal pathways linking this circuit with attention and default mode networks (DMN) are characteristic of MDD [8] (for review [2, 7]). Depressed patients who do not remit on commonly prescribed pharmacotherapies show dorsolateral prefrontal cortex (DLPFC) hypoactivation along with hypoconnectivity between the DLPFC and anterior cingulate cortex [9]. Corresponding frontoparietal attention network hypoconnectivity is also observed in MDD [10, 11] and correlated with behavioral indices of poor attention, such as false alarm errors on cognitive testing, in related anxiety disorders [12]. Some degree of DMN dysfunction is observed in persistently unwell MDD patients [13] and can identify MDD patients who do not remit on antidepressants [14]. Reflecting the close interplay of cognitive control and attention networks, pharmacoresistant patients with MDD are also characterized by impaired connectivity of the DLPFC and precuneus component of the attention network [15]. As an interposed area involved in the interactive effects of cognitive control and other circuits, subgenual anterior cingulate cortex (sgACC) impairments are also thought to exacerbate broader circuit dysfunction in MDD (for review [10]). Pharmacoresistant MDD patients show persistent hypoactivation and connectivity involving the sgACC [16] in analyses of brain metabolism using positron emission tomography.

Repetitive transcranial magnetic stimulation (rTMS, hereafter TMS) was cleared by the U.S. Food and Drug Administration (FDA) for pharmacoresistant MDD in 2008 and has become an important treatment option in clinical settings. While the putative therapeutic mechanism of TMS remains under study, recent neuroimaging studies provide insight into brain activity changes associated with therapeutic TMS of the DLPFC. Neuroimaging studies of TMS in both healthy subjects and in MDD have focused mostly on circuits probed during resting conditions. In healthy subjects our prior work has shown that DLPFC stimulation induces an inverse correlation between resting connectivity of the DLPFC (middle frontal gyrus) and the medial frontal region of the DMN [17] as expected for flexible circuit organization. Neuroimaging studies implicate pre-stimulation baseline DLPFC-sgACC connectivity in the mechanisms of clinical action of TMS [18, 19]. In 13 patients with MDD, more intact negative (i.e., “anticorrelated”) DLPFC-sgACC resting state functional connectivity prior to DLPFC stimulation was associated with superior amelioration of clinical symptoms [18]. In a small subset of controls and two patients scanned post-TMS, individual differences in DLPFC-sgACC connectivity were highly reproducible [19]. These findings suggest that suppression of the subgenual anterior cingulate cortex via DLPFC stimulation may be an antidepressant mechanism of TMS, and that baseline connectivity is a viable imaging biomarker to optimize TMS at the individual level. When imaged after TMS, responders (*n*=5/12) showed improvement in the negative connectivity of DLPFC and sgACC, whereas non-responders (*n*=7/12) did not [20]. In a complementary study, 17 MDD patients were found to show attenuation of abnormally positive sgACC-DMN connectivity, along with reduced DLPFC to medial prefrontal connectivity, but not of the DLPFC and sgACC [21]. However, in this latter study, TMS-related connectivity changes were not associated with clinical improvement. Most recently, Weigand et al. [22] demonstrated that sgACC-DLPFC connectivity could predict clinical response to TMS; this study incorporated two datasets, inclusive of 25 participants who received unblinded TMS, and 16 participants who received sham stimulation and open-label stimulation from Taylor et al. [23]. Together, findings to date suggest that TMS selectively modulates functional connectivity both within and between the cognitive control network and interconnected regions of the frontal cortex and DMN, and that modulation of these interactions by the sgACC may play an important mechanistic role in predicting the effect of TMS on alleviating depression. The results also highlight the need for systematic investigation using imaging biomarkers in samples with greater power for statistical inference.

Drawing on this evidence, a premise of our study is that TMS of the DLPFC will have antidepressant efficacy via direct effects on cognitive control processes that contribute to regulatory functions and that involve interactions with attention and default mode networks.

Despite the wide scale adoption of TMS, we still lack mechanistically-driven biomarkers designed to identify who is most likely to respond, and why; these measures are crucial for broader adoption of TMS and are possible with near-term discoveries. In our recent multisite trial of TMS in pharmacoresistant depression [24], standard clinical measures did not predict remission [25]. While there are relatively few side effects from TMS as compared to other neuromodulation techniques, undergoing a full course of this treatment when it will ultimately not lead to remission can be discouraging for the patient and psychiatrist, prolongs suffering and is economically inefficient. For these reasons, there is an urgent need for well-powered multisite clinical trials that advance a biomarker-driven approach to identifying which patients will benefit from TMS and through which mechanisms. Furthermore, existing rubrics can be immediately used to translate observed findings into clinical practice [26]. Our findings will, more broadly, also lay important foundations for the systematic experimental manipulation of stimulation protocols and parameters in future mechanistic trials.

Our study objective is to systematically evaluate cognitive control network connectivity and behavior as response biomarkers for the effect of TMS in pharmacoresistant MDD, and the extent to which connectivity and behavior are predictive of clinical symptoms, function, and suicidality outcomes. We strive to meet this objective by exploring the following aims:

### Aim 1

To evaluate a response biomarker of the effect of TMS on promoting cognitive control. We will assess whether activation and functional connectivity of the DLPFC-anchored cognitive control network, and interactions with sgACC, attention and default mode regions involved, change in a session (akin to dose)-dependent manner. Our broad hypothesis is that functional connectivity will change from the pre-treatment baseline to reassessment early after commencement of TMS (targeting 5 sessions) and later, post- completion of treatment (targeting 30 sessions). Related, we hypothesize that the early change will be most pronounced for patients with intact connectivity at baseline; later change post-treatment will be observed for those with more impaired baseline connectivity. First, we will address the mechanistic question of whether early changes in circuit connectivity are necessary, if not sufficient, for subsequent circuit and clinical changes observed post-TMS. Second, we will systematically test whether the extent of change in connectivity is related to the extent of dysfunction at the pre-stimulation baseline. Third, we will probe whether observed changes in connectivity increase as a session-dependent function of the total number of TMS sessions. In addressing these issues, we will incorporate a methodological technique to quantify the site of DLPFC stimulation with anatomical precision.

### Aim 2

To assess whether the extent of change in a DLPFC cognitive control network connectivity response biomarker is related to corresponding change in behavioral performance. Our broad hypothesis is that the extent of connectivity change will be related to the extent of change in behavioral performance, and that this relationship will be most pronounced for patients with relatively intact connectivity at baseline; later change will be observed in patients with more impaired baseline connectivity.

### Aim 3

To identify if pre-treatment functional connectivity of the DLPFC cognitive control network, involving interactions with the sgACC and regions of the attention and default mode networks, and behavioral performance, along with early change in connectivity and behavior, are predictive biomarkers of clinical outcome. Our hypothesis is that baseline connectivity and behavior, and early and later changes in these measures will predict who at post-treatment have the greatest change in symptom severity, suicidality, and quality of life.

## Methods and design

### Recruitment and screening

To achieve a target enrollment size of 100, we aim to recruit 125 veterans with pharmacoresistant MDD across sites participating in the VA Clinical TMS Program. This recruitment number (*n*= 125) allows for an anticipated 20% drop out rate of ~ 25 veterans, while still ensuring sufficient statistical power to address the aims of the study. To meet this target, we will recruit approximately 30 veterans per site. We set a minimum of 25 veterans and a maximum of 40 per site to ensure that we retain equivalent site representation. Given the complex nature of the veteran sample, the primary diagnosis of MDD may be comorbid with other disorders, including post-traumatic stress disorder (PTSD).

Veterans will be recruited from four initial preexisting clinics within the Clinical TMS Program: Palo Alto VA Medical Center, White River Junction VA Medical Center, Minneapolis VA Medical Center, and Providence VA Medical Center. All referral sources agree to maintain their clinical relationship with the veterans and attempt to keep medication regimen as stable as possible. Every veteran seen in the TMS Clinical Program will be invited to participate in this study as long as they meet the eligibility criteria. See Table 1 for a summary of the eligibility criteria. The invitation to participate will be extended at the end of the veterans’ initial TMS clinical evaluation with the attending TMS physician. Each veteran’s research involvement is expected to last throughout the course of their clinical TMS treatment.

**Table 1:** Inclusion and Exclusion Criteria

***Inclusion Criteria***	
● Ages 18 years and older	
● Meets DSM-5 criteria for MDD (as documented by the TMS physician)	
● Meet study criteria for pharmacoresistance in accordance with the Clinical TMS Program (i.e. failed at least one antidepressant in the current episode)	
● Ability to obtain a motor threshold (MT) prior to the start of treatment	
● Stable medical conditions and ability to maintain stability on current medication regimen for the duration of treatment	
● Ability to participate in a daily treatment regimen	
● Able to read, verbalize understanding, and voluntarily sign the Informed Consent Form prior to participating in any study-specific procedures or assessments	
***Exclusion Criteria***	
● History of seizure disorder	
● Structural or neurologic abnormalities present or in close proximity to the treatment site	
● History of brain surgery	
● Pacemaker or medical infusion device (unless MRI compatible)	
● History of traumatic brain injury within 60 days of the start of treatment	
● Severe or uncontrolled alcohol or substance use disorders	
● Active withdrawal from alcohol or substances	
● Implanted device in the head	
● Metal in the head	
● Severe impediment to vision, hearing and/or hand movement, likely to interfere with ability to complete the assessments, or unable and/or unlikely to follow the study protocols	
● Lifetime history of bipolar I disorder	
● Inability to speak, read or understand English	
● Plans to move out of the area during the study period	
● Clinician and/or Investigator discretion for clinical safety or protocol adherence	

All veterans referred to the TMS Clinic are evaluated by a physician familiar with neuromodulation techniques who determines their eligibility for treatment. Veterans deemed suitable for TMS treatment are then screened by a research coordinator to determine their study eligibility based on the inclusion and exclusion criteria above

### Data collection procedures

**Fig. 1:**
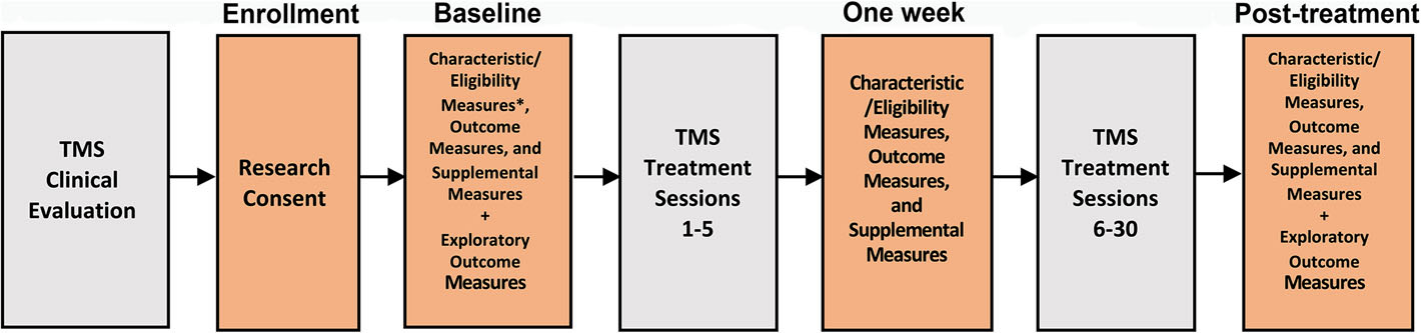
Procedural Diagram. All veterans that consent to research and meet eligibility criteria will follow this procedural diagram. Veterans will attend four research assessment sessions (highlighted in orange). ‘Baseline’ refers to the research assessment session prior to commencement of TMS treatment, ‘1 Week’ to the assessment session undertaken after a target of five sessions of TMS treatment, and ‘Post-treatment’ to assessment sessions undertaken following the completion of the full course of TMS treatment which is typically 30 treatment sessions.*The MINI-7 is the only Sample Characteristic and Eligibility Measure that will be given at the Enrollment Visit instead of the Baseline research session

Figure 1 details the study diagram of veterans undergoing both clinic sessions and research assessments. ‘Baseline’ refers to research assessments prior to commencement of TMS treatment, ‘1 Week’ refers to assessments undertaken after sessions completed in the first week, typically after five sessions, and ‘Post-treatment’ to assessments undertaken following the completion of the full course of TMS treatment, which is typically 30 sessions. Research assessments will occur at both VA and VA-affiliated academic medical centers and via remote procedures as required. Transportation between research visits is provided for veterans as required. Each research assessment consists of different data collection procedures. Collected data will include functional neuroimaging, cognitive and behavioral measures, clinical measures, and complementary neuropsychological measures. Each measure and its function are described in further detail below. To ensure data validity and reliability, the protocol is designed to complete assessments within specific time-windows. For a visual representation of data collection time windows, see Table 2.

**Table 2:** Data Collection Windows for Research Assessment Sessions

	Baseline	1 Week	Post-treatment
**Standard**	< 72 h	< 24 h	< 72 h
**Acceptable**	< 10 days	< 72 h	< 10 days
**Unusable**	> 14 days	> 96 h	> 14 days

The Standard designation denotes the preferred data collection window for each research assessment session. Data will still be accepted if obtained within the Acceptable time window. Data obtained during the Unusable window will not be used. Windows refer to the following: Baseline for assessments completed prior to commencement of TMS, 1 Week for assessments completed after five sessions of TMS and Post-treatment for assessments completed after 30 sessions of TMS. Standard and Acceptable window data will be treated the same way for data analysis purposes

### Measures of sample characteristics and eligibility measures

Measures of Screening and Eligibility will help establish veterans’ ability to participate in research and gather diagnostic clarification. See Table 3 for a detailed summary of screening and eligibility measures. A majority of our self-report measures are administered using a computerized format, suited to remote assessment when required, and recorded within the REDCap database. Raw scores are automatically transformed to standard scores within the REDCap system as appropriate. All self-report assessments will be administered via computer or tablet. Assessments that are not computerized will be administered and scored by a research coordinator or neuropsychology trainee via clinically developed teleneuropsychology procedures consistent with published guidelines [27–30].

1. **MINI International Neuropsychiatric Interview- 7th Edition (MINI-7)**: This measure is an eight-item questionnaire. The 7th edition of the MINI is a derivative of the original MINI used in conjunction with the DSM-III-R [31]. The MINI-7 will provide further psychiatric diagnostic clarification utilizing the DSM-5, administered by research personnel.
2. **Self-Administered Comorbidity Questionnaire (SCQ)**: Veterans will complete this measure on a desktop computer or tablet. This task assesses up to 15 commonly occurring psychiatric and general medical disorders [32]. Ratings assess the presence of the disorder, whether or not veterans are receiving treatment for the disorder, and whether or not the disorder is related to functional limitations.
3. **Alcohol Use Disorders Identification Test (AUDIT):** The AUDIT includes 11 questions developed for the identification of individuals with alcohol use problems [33].
4. **Drug Use Disorders Identification Test (DUDIT):** The DUDIT includes 11 questions that assess the presence of drug-related problems [34]. This measure was developed as a parallel instrument to the AUDIT and will be administered via computer or tablet.
5. **PTSD Checklist for DSM-5 (PCL-5) and PCL-WEEKLY**: The PCL-5 is a 20-item measure that assesses DSM-5 symptoms of PTSD [35]. The PCL-5 and PCL-Weekly have identical questions, but different instructions. The PCL-5 will be administered at Baseline and at the Post-treatment assessment while the PCL-WEEKLY will be administered weekly to align with the standardized assessment scheduled for the Clinical TMS program.
6. **Life Events Checklist for DSM-5 (LEC-5):** The LEC-5 is a 17-item measure that screens for potentially traumatic events in a respondent’s lifetime [36]. There is no formal scoring protocol or interpretation, other than identifying whether a person has experienced one or more traumatic events.
7. **Advanced Clinical Solutions (ACS)**: A measure estimating premorbid ability using the Test of Premorbid Functioning (TOPF) word reading list [37]. This measure will only be administered at Baseline.

**Table 3:** Data Collection Summary for Sample Characteristics and Eligibility Measures

	Enrollment	Baseline	1 Week	Post-treatment
Consenting Process (for study, fMRI, & testing)	X			
**Measures of Sample Characteristics and Eligibility**				
MINI International Neuropsychiatric Interview- 7 (MINI-7)	X			
Self-Administered Comorbidity Questionnaire (SCQ)		X	X	X
Alcohol Use Disorders Identification Test (AUDIT)		X	X	X
Drug Use Disorders Identification Test (DUDIT)		X	X	X
PTSD Checklist for DSM-5 (PCL-5)		X		X
PCL-5 Weekly Questionnaire			X	
Life Events Checklist for DSM-5 (LEC-5)		X		X
*Estimates of Intellectual Premorbid Functioning*				
Advanced Clinical Solutions (ACS; Test of Premorbid Functioning)		X		

Data collection occurs at 4 time points as shown above. Enrollment and Baseline research sessions occur prior to the commencement of TMS treatment session 1. The 1 Week research session occurs after completion of the first TMS treatment sessions within the first week, targeting the first 5 TMS sessions. The Post-treatment research session occurs after completion of TMS treatment, targeting 30 TMS sessions

## Functional neuroimaging protocol for assessing circuit function (aim 1)

The neuroimaging protocol will be standardized across sites (refer to **Harmonization of Scanners** below). Neuroimaging will be acquired using 3 T GE Discovery MR750 UHP (GE Healthcare, Milwaukee, WI, USA) and 3 T Siemens Magnetom Prisma Fit (Siemens Medical Solutions USA, Malvern, PA, USA) scanners at VA-affiliated institutions including Stanford University, Dartmouth-Hitchcock, Brown University, and the University of Minnesota. We will perform functional and structural MRI scans at the Baseline, 1 Week and Post-treatment visits. For functional imaging, we will administer both task-based (GoNoGo and N-Back cognitive control tasks) and resting state protocols.

### Task-based fMRI and resting state

**Fig. 2:**
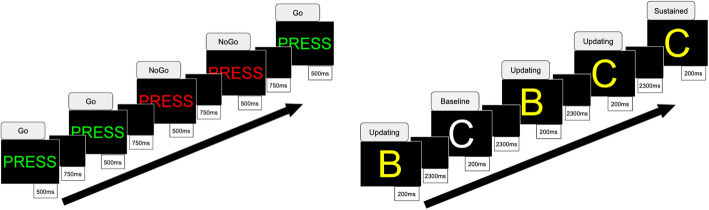
GoNoGo and N-Back Tasks. A visual summary of the GoNoGo paradigm [38] (left) and the N-Back working memory paradigm [39] (right) for probing the cognitive control network during functional neuroimaging

1. **GoNoGo**. We will use the GoNoGo task (depicted in Fig. 2) to assess response inhibition and cognitive control. Behavior is assessed in terms of “False alarm” NoGo errors and reaction time for Go stimuli. This task is well normed across nine decades [40], and has sound test-retest reliability, including parallel forms for repeat testing. It has been shown to robustly elicit inhibition errors in MDD and PTSD [15, 41]. In the GoNoGo task, veterans respond via button press as quickly and accurately as possible to Go stimuli (the word “Press” in green) and withhold responses to NoGo stimuli (the word “Press” in red). There are 180 Go and 60 NoGo stimuli (ratio 3:1), presented pseudorandomly with duration of 500 ms and jittered interstimulus interval of 750 ms.
2. **N-Back Working Memory**. The N-Back working memory task (depicted in Fig. 2) has been used previously to probe working memory maintenance and sustained attention functions in depression [42]. Stimuli are presented under three conditions: 30 sustained attention stimuli in which yellow letters appear twice in a row and veterans respond to the consecutive yellow letter; 50 working memory stimuli in which yellow letters appear randomly and not consecutively and veterans are required to maintain and update working memory without responding to the letters; and 40 perceptual baseline stimuli in which to-be-ignored white letters are presented as a perceptual contrast to yellow letters. Working memory stimuli are not presented in a design that manipulates different levels of working memory demand.
3. **Resting State.** Veterans will be instructed to stare at a white cross on a black background. During this time, their eyes will be monitored using an eye tracker by the study coordinator to ensure they are not asleep.

### Acquisition sequences and details

The MRI protocol starts with scanner’s default localizer to locate the brain. Next, spin-echo fieldmaps are acquired with two different phase encodings to help with distortion correction. The resting state and N-Back fMRI acquisitions are calibrated with a single-band reference image and then immediately followed by a multi-band sequence (SMS factor = 6). The resting state sequence is split into two opposing phase encoding directions to average the effects of distortion. The GoNoGo task uses a single band sequence with a TR of 2 s. Finally, the protocol ends with a T1 sequence using prospective motion correction (PROMO). These parameters yield relevant activations as shown by the group level analyses in the independently funded Human Connectome Project for Disordered Emotional States [42]. The parameters are summarized below.

1. **Spin-echo fieldmaps**: FA = 90, field of view = 216 × 216 mm, 3D matrix size = 90 × 90 × 60, angulation to anterior commissure - posterior commissure (AC-PC) line, phase encoding = AP and PA, voxel size = 2.4 mm isotropic.
2. **Single-band calibration**: FA = 90, field of view = 216 × 216 mm, 3D matrix size = 90 × 90 × 60, slice orientation = axial, angulation to AC-PC line, phase encoding = AP and PA, number of volumes = 4, voxel size = 2.4 mm isotropic.
3. **Multi-band fMRI**: TE = 30 ms, TR = 0.71 s, FA = 54, acquisition time = 5:12 (Resting State × 2), 5:08 (N-Back), field of view = 216 × 216 mm, 3D matrix size = 90 × 90 × 60, slice orientation = axial, angulation to AC-PC line, phase encoding = PA and AP, number of volumes = 428 (Resting State), 422 (N-Back), multiband factor = 6, voxel size = 2.4 mm isotropic. The low multiband factor (6) and larger voxel size (2.4 mm) increase the signal to noise ratio, especially in our subcortical structures of interest [43].
4. **Single-band fMRI**: TE = 27.50 ms, TR = 2 s, FA = 77, acquisition time = 5:08 (GoNoGo), field of view = 222 × 222 mm, 3D matrix size = 74 × 74 × 45, slice orientation = axial, angulation to AC-PC line, phase encoding = PA, number of volumes = 151, voxel size = 3 mm isotropic.
5. **T1-weighted anatomical**: TE = 3.8 ms, TR = 3 s, FA = 8, field of view = 256 × 256 mm, 3D matrix size = 320 × 320 × 230, slice orientation = sagittal, angulation to AC-PC line, motion correction = PROMO, voxel size = 0.8 mm isotropic; parallel imaging technique = GRAPPA/ARC.

### Localization of stimulation site

In order to localize the site of stimulation with respect to our image analyses, we will use a PinPoint® for Small Field of View Imaging 187 (Beekley Medical, Bristol, CT, USA) MR-opaque gel capsule over the F3 site during scanning. This approach will allow for the stimulation target position (F3) to be ‘marked’ on the structural scan relative to the ideal resting state target as has been previously demonstrated [22]. Some evidence suggests that stimulation of the optimal BA46 region within the DLPFC with the most anticorrelation to sgACC produces larger effects on clinical measures [18, 22], and we will test this in exploratory analyses.

### Quality control and motion correction

We will restrict head motion during acquisition using foam inserts. We will record motion for subsequent correction using the PROMO [44] system on the GE scanner and the fMRI Integrated Real-time Motion Monitor (FIRMM) [45] on the Siemens scanners. For post-acquisition, we will implement the full fBIRN quality control (QC) metrics used in the Human Connectome Project protocol [42] and that are established at the Stanford coordinating site. In addition, we will implement scripts developed by Stanford personnel for additional motion scrubbing [46–48]. These scripts are designed to remove the variance of specific TRs associated with extreme movement as follows: 1) from one volume to the next (calculated by as the sum of the absolute values of the differentiated realignment estimates) and, 2) changes in BOLD signal from one volume to the next (as indexed by the temporal derivative of RMS variance over voxels) implemented with SPM’s time series difference analysis toolbox [49].

### Harmonization of scanners

Neuroimaging will be acquired using 3 T GE Discovery MR750 UHP and 3 T Siemens PrismaFit scanners. To ensure consistent data acquisition, much thought was put into parameter harmonization. First the fMRI sequence was developed on Stanford’s GE scanner and consisted of single-band, single-band reference, multiband, spin-echo fieldmaps, and T1 images. Next the sequence parameters were shared between GE and a Siemens scanner with close attention paid to idiosyncrasies between the GE and Siemens systems. Limitations on both systems required modifying the protocol so that parameters would match. Parameter comparisons were carried by checking both the printouts and comparing information found in the dicom header. The remaining Siemens sites received the protocol by sharing “.exar1” files. The reliability of the harmonization procedures will be assessed by acquiring test data at all sites, consisting of repeat scanning of the same subjects and a phantom. This data will be used to determine inter- and intra-site variation. Phantoms will be scanned with the fMRI SMS sequence for 8 min at each site to monitor quality through SNR metrics on a monthly basis [43, 50]. Additionally, the phantom acquisitions will be used to reduce inter-scanner variability [40]. We will keep a record of which participants are required, in response to public health guidance, to wear masks during MRI sessions, along with a record of which session and which sites.

## Neurobehavioral protocol for assessing performance (aim 2)

Neurobehavioral measures will be assessed across three research sessions. For a summary of the administration schedule for primary and secondary outcome measures, see Table 4.

**Table 4:** Data Collection Summary for Primary and Secondary Outcome Measures

	Enrollment	Baseline	1 Week	Post-treatment
**Outcome Measures**				
**Primary Outcomes - Neuroimaging (Aim 1)**				
*Functional MRI*				
GoNoGo Task		X	X	X
N-Back Working Memory Task		X	X	X
Resting State		X	X	X
*Structural MRI*				
T1 Anatomical		X		X
**Secondary Outcomes - Cognitive-Behavioral Measures (Aim 2)**				
CNS Vital Signs Battery (SDC, Stroop, SAT, CPT Subtests)		X	X	X
Webneuro Abbreviated Version (GoNoGo and N-Back Subtests)		X	X	X
**Secondary Outcomes - Clinical Outcome Measures (Aim 3)**				
Quick Inventory of Depressive Symptoms (QIDS)- Self Report Form		X	X	X
Veterans’ RAND 36-item Health Survey (VR-36)		X	X	X
Columbia-Suicide Severity Rating Scale (C-SSRS)		X	X	X

Data collection occurs at 4 time points as shown above. Enrollment and Baseline research sessions occur prior to the commencement of TMS treatment session 1. The 1 Week research session occurs after completion of the first TMS treatment sessions within the first week, targeting the first 5 TMS sessions. The Post-treatment research session occurs after completion of TMS treatment, targeting 30 TMS sessions

## Measures of cognitive performance

### CNS vital signs

This computerized neurocognitive test battery comprises 10 subtests measuring different aspects of cognitive functioning [54]; however, for the purposes of this study we will utilize and administer eight subtests using a desktop computer. Four of these subtests will be utilized to future investigate the cognitive control circuit, while four will be used as supplemental measures to control for potential confounding variables, such as motor speed. Psychometric properties including test-retest reliability have been established for these subtests [55]. The program software will automatically score and standardize the raw scores upon test completion.

### WebNeuro

Computerized tests of cognitive control performance will be measured offline using WebNeuro [51–53]. These tasks will be executed on a computer by the veteran at the Baseline, 1 Week, and Post-treatment research assessment sessions. The software used to run the tasks incorporates standardized task instructions. Psychometric properties have been established for each of these tests, including norms, construct validity, validation against traditional neuropsychological tests evaluating equivalent functions, test-retest reliability, and consistency across cultures [2]. For each test, we will record accuracy and reaction time.

1. **N-Back Task/Continuous Performance Test**: This is a measure of sustained attention. A series of 125 similar looking letters (B, C, D, or G) are presented to the veteran on the computer screen for 200 msec with an interval of 2.5 s between each letter. If the same letter appears twice in a row, the veteran is required to press the spacebar. There are 85 nontarget letters and 20 target letters (i.e. repetitions of the previous letter).
2. **GoNoGo Task:** A word (press) is frequently presented in the color *green* (Go) and infrequently in the color *red* (NoGo). The veteran is asked to respond with a keypress when the word is presented in green and inhibit a keypress when it is presented in red. Inhibition is assessed with omission of keypress responses when the word ‘press’ is red.
3. **Symbol Digit Coding (SDC):** This test consists of serial presentations of screens, each of which contains a bank of eight symbols above and eight empty boxes below. The veteran types in the number that corresponds to the symbol that is highlighted.
4. **Stroop Test:** The Stroop test has three parts which measure simple and complex reaction time, inhibition, frontal skills, and processing speed.
5. **Shifting Attention (SAT):** This test is a measure of ability to shift from one instruction set to another quickly and accurately. Veterans are instructed to match geometric objects either by shape or by color.
6. **Continuous Performance (CPT):** This test is a measure of vigilance or sustained attention. The test subject is asked to respond to the target stimulus “B” but not to any other letter. The stimuli are presented at random.

## Clinical measures for assessing functional outcomes (aim 3)

Self-report measures will be used to assess clinical change in depression, medical history, and suicide risk. These measures are administered using a computerized format, suited to remote assessments when required, and recorded within the REDCap database. Raw scores are automatically transformed to standard scores within the REDCap system as appropriate.

### Depression

Depression severity based on DSM-5 criteria will be assessed using the Self-Report version of the Quick Inventory of Depressive Symptoms (QIDS-SR) [56].

### Medical history

We will use the Veterans’ RAND 36-item Health Survey (VR-36) to assess eight dimensions of function relevant to physical and mental health [57, 58] including role limitations due to physical problems, bodily pain, general health perceptions, vitality, and social functioning, and role limitations due to emotional problems and mental health.

### Suicide risk

Suicidality will be assessed using the Columbia-Suicide Severity Rating Scale (C-SSRS), a semi-structured clinical interview used to measure suicidal behavior [59]. This assessment requires additional follow-up questions based on the veterans’ responses; thus, this task will be administered via computer or tablet with the guidance of the administrator.

#### Complementary outcome measures

For a summary of complementary measures, see Table 5.

**Table 5:** Data Collection Summary for Complementary Outcome Measures

	Enrollment	Baseline	1 Week	Post-treatment
**Supplemental Cognitive-Behavioral Measures (Aim 2)**				
CNS Vital Signs (VBM, VIM, FTT and POET Subtests)		X	X	X
**Exploratory Cognitive-Behavioral Measures (Aim 2)**				
*Executive Functioning*				
Trail Making Test A and B ^a^		X		X
Wechsler Adult Intelligence Scale-4th edition (WAIS-IV): Coding Subtest ^a^		X		X
Wechsler Adult Intelligence Scale-4th edition (WAIS-IV): Digit Span Subtest		X		X
Delis-Kaplan Executive Function System (D-KEFS): Color-Word Interference Subtest ^a^		X		X
Delis-Kaplan Executive Function System (D-KEFS): Verbal Fluency Subtest- Standard Form		X		
Delis-Kaplan Executive Function System (D-KEFS): Verbal Fluency Subtest- Alternate Form				X
*Verbal and Nonverbal Memory Functioning*				
California Verbal Learning Test-Third Edition (CVLT-III)- Standard Form		X		
California Verbal Learning Test-Third Edition (CVLT-III)- Alternate Form				X
Brief Visuospatial Memory Test-Revised (BVMT-R): Form 1 ^a^		X		
BVMT-R: Alternate Form 2 ^a^				X
*Motor Functioning*				
Grooved Pegboard ^a^		X		X

This table describes the schedule of each complementary test. Data collection occurs at 4 time points as shown above. Enrollment and Baseline research sessions occur prior to the commencement of TMS treatment session 1. The 1 Week research session occurs after completion of the first TMS treatment sessions within the first week, targeting the first 5 TMS sessions. The Post-treatment research session occurs after completion of TMS treatment, targeting 30 TMS sessions. ^a^These assessments may be altered or not administered if teleneuropsychological administration is conducted

##### Supplemental cognitive-behavioral measures

As described above (see CNS Vital Signs), four subtests of the CNS Vital Signs Battery will be used as supplemental measures to control for potential confounding variables, such as motor speed, memory, and perception.

1. **Verbal Memory (VBM):** A measure of recognition memory for words. Fifteen words are presented, one by one, on the screen every two seconds. For immediate recognition, the veteran must identify those words nested among fifteen new words. Then, after six more tests, there is a delayed recognition trial.
2. **Visual Memory (VIM):** Measures recognition memory for figures or shapes. Fifteen geometric figures are presented, one by one, on the screen. For immediate recognition, the veteran must identify those figures nested among fifteen.
3. **Finger Tapping (FTT):** This test requires subjects to press the spacebar with their right index finger as many times as they can in ten seconds. They do this once for practice, and then there are three test trials. The test is repeated with the left hand.
4. **Perception of Emotions (POET):** Measures how well a subject can perceive and identify specific emotions.

##### Exploratory neurocognitive measures

A brief neuropsychological evaluation will be administered for the purpose of future clinical translation. We will attempt to administer these assessments in-person using an internally certified neuropsychology trainee or research coordinator. However, under public health circumstances in which assessments may have to be conducted remotely, we will use teleneuropsychological administration. Teleneuropsychological administration is considered an appropriate alternative for several cognitive measures that will be used within this study [28, 60–62] and will be guided by best practices [27, 29, 30, 63, 64]. Certification requires attending a training seminar developed by a licensed clinical neuropsychologist, being cleared by the neuropsychologist or the appropriate proxy who is supervised by the neuropsychologist to administer each measure, and attending quarterly supplemental refresher courses. The cognitive measures were selected in order to capture general cognitive functioning within the specific domains of memory, attention, language, executive functioning, motor functioning, and visuoperceptual abilities. The neuropsychological battery will be altered to use alternate forms when available depending on the assessment session. Specifically, veterans will be given alternative test stimuli at the Post-treatment research session in order to help alleviate concerns regarding practice effects and test-retest reliability. Published reliable change indices will be utilized to detect clinically meaningful change for tests where alternate test forms are unavailable. Table 5 provides a summary of additional exploratory measures administered in this study.

Administrators will use test-specific normative data to convert veterans’ raw scores into standardized scores. Each site will also attempt to use the same testing administrator at both Baseline and Post-treatment research sessions in order to avoid individual differences in test administration and scoring. Sites will be encouraged to have a second person score each measure to ensure accuracy.

1. ***Executive Function***
  - **Trail Making Test A and B**: A measure of processing speed, sequencing, mental flexibility and visual–motor skills [65]. Standardizing raw scores will be accomplished using the Revised Comprehensive Norms for an Expanded Halstead-Reitan Battery: Demographically Adjusted Neuropsychological Norms for African American and Caucasian Adults [66]. We will use the reliable change indices from Dikmen et al. [67]. If teleneuropsychological administration is conducted, Oral Trails [68] may be used as a modification to the Trail Making Test A and B.
  - **Wechsler Adult Intelligence Scale-4th edition (WAIS-IV) – Selected Subtests**: A measure of processing speed (i.e., Coding) and simple and complex auditory attention (i.e., Digit Span) [37] will be administered. Standardized scores and reliable change indices will be obtained using the normative data from the WAIS-IV Examiner’s and Technical Manuals. If teleneuropsychological administration is conducted, Coding may not be administered.
  - **Delis-Kaplan Executive Function System (D-KEFS) – Selected Subtests**: A measure of visual processing speed and cognitive control will be administered (i.e., Color-Word Interference). A measure of lexical fluency, semantic fluency, and executive control will also be administered (i.e., Verbal Fluency Subtest (VF), Standard and Alternate Form) [69]. The VF-Standard Form will be administered at Baseline, while the VF-Alternate Form will be given at the Post-treatment Visit. Standardized scores and reliable change indices will be derived from the D-KEFS Examiner’s and Technical Manuals. If teleneuropsychological administration is conducted, slight modifications will be used for the Color-Word Interference subtest.
2. ***Verbal and Nonverbal Memory Function***
  - **California Verbal Learning Test-Third Edition (CVLT-III)**- Standard and Alternate Form: The CVLT-III is a 16-item list assessment that measures verbal learning, memory, and recognition [70]. The Standard Form will be administered during the Baseline Visit, while the Alternate Form will be administered during the Post-treatment Visit. Normative data from the CVLT-III Examiner’s Manual will be used for standardizing raw scores.
  - **Brief Visuospatial Memory Test-Revised (BVMT-R), Form 1 and Form 2**: The BVMT-R is an assessment used to measure visuospatial learning and memory [71]. Form 1 will be administered at Baseline and Form 2 will be given at the Post-treatment Visit. Standardized scores will be derived from the BVMT-R Examiner’s Manual. If teleneuropsychological administration is conducted, slight modifications will be made for this measure.
3. ***Motor Function***
  - **Grooved Pegboard**: The Grooved Pegboard is a measure of eye-hand coordination and motor speed and often used to localize deficits [65]. Standardized scores will be derived from the Revised Comprehensive Norms for an Expanded Halstead-Reitan Battery: Demographically Adjusted Neuropsychological Norms for African American and Caucasian Adults [66] and reliable change indices will also be used [67]. If teleneuropsychological administration is conducted, Grooved Pegboard may not be administered.

## Regular care TMS protocol within the partner clinical TMS program of the VA

Veterans will receive TMS treatment as part of their regular clinical care within the VA. Because we will utilize the umbrella VA Clinical TMS Program, we can ensure that the parameters received by veterans are uniform. Treatment will be delivered using the Magstim Rapid 2 or Magstim Horizon Performance devices (The Magstim Company Ltd., Whitland, United Kingdom).

At the time of study design, current clinical practice standards for MDD indicate one session of TMS per day five times per week for 6 weeks. This protocol assumes a magnetic field intensity of 120% of motor threshold at a frequency of 10 Hz at the left DLPFC stimulation site. Treatment sessions take approximately 37.5 min resulting in 3000 pulses per treatment session delivered based on the following treatment parameters: train duration of 4 s, inter-train interval of 26 s, and 75 trains. Stimulation targets are reached through anatomical landmarks using the Beam-F3 method [72].

Depending on veteran treatment response and physician discretion, the standard TMS treatment protocol may be subject to change throughout the treatment course.

## Statistical analysis

### Power calculation

The sample size was calculated based on a conservative scenario in which we estimate a main effect for TMS on targets of interest of small effect size (Cohen’s d of approximately 0.25), and use a within-subjects linear model with session as a repeated measure, at least one moderator of interest (extent of baseline connectivity dysfunction) and three covariates. It is possible that effect sizes are larger. Dependent measures are connectivity, behavior, and self-report measures (3 measurement domains), and change in these measures, assessed in separate models. With an alpha level of 0.05 (two-tailed), power of 0.875 and an anticipated correlation of 0.5 for repeated sessions we require at least 98 veterans. To target 100 veterans and allow for the potential for dropout over the 3 sessions we aim to recruit 125 veterans. If a greater effect size is obtained, this would lead to greater power.

### Data analysis plan

We will pursue a stepwise analysis plan that starts with a focus on our a priori regions of interest and builds to a model based on machine-learning of these regions, and then an exploratory phase, as follows:

For *Aim 1*, we will quantify functional connectivity in the resting and task conditions by computing correlation coefficients between the a priori regions of interest and converting these coefficients to Fisher Z scores. In task-evoked conditions we will also use gPPI to quantify connectivity incorporating the task contrast. Multivariate linear mixed models will be used to test the hypothesis that extent of connectivity is a response biomarker determining extent of post-treatment change in both connectivity and in clinical measures after 1 Week of treatment and Post-treatment (within-subjects). We will include both binary between-subjects and continuous moderators to assess whether veterans with intact pre-treatment connectivity show connectivity change after early sessions whereas veterans with impaired pre-treatment connectivity show change after completion of sessions. Correlational analyses will be used to test whether the extent of early change is proportional to the extent of baseline connectivity impairment.

For *Aim 2*, we will use the connectivity values established under Aim 1. Linear mixed models, with behavioral measures included as dependent variables, will be employed to test the hypotheses that extent of connectivity relates to extent of behavioral performance and that change in connectivity predicts change in behavioral performance (within subjects). We will include binary and continuous moderators to test if these relationships differ as a function of degree of baseline connectivity dysfunction after 1 Week and after Post-treatment sessions.

For *Aim 3*, we will again use the connectivity values established under Aim 1. Linear mixed models, with symptom, function, and suicidality measures included as dependent variables, will be employed to test the hypotheses that extent of baseline connectivity predicts severity of symptoms, functional disability and suicidality and that change in connectivity predicts changes in symptoms, function, and suicidality (within subjects). We will include binary and continuous moderators to test if these relationships differ as a function of degree of baseline connectivity dysfunction for after the 1 Week and Post-treatment sessions.

### Interactions, covariates and cross-validation

Under each of these aims, we will evaluate if the interaction of the DLPFC-anchored cognitive control network with resting attention and default mode networks, and the sgACC, further moderates these relationships. In each analysis, we will model sex, medication, medication change, comorbidity, substance use, and premorbid function as covariates. We will employ cross-validation techniques, as used in our prior pharmacotherapy and imaging trials [14, 73, 74], to assess the rigor of our inferences.

### Exploratory analyses

We will pursue the following additional exploratory options: 1) covariation due to stimulation site quantified by our gel capsule method, 2) canonical correlations to quantify dimensional relations between connectivity, behavior and symptom, function and suicidality measures, 3) predictive regression models to further interrogate our hypotheses that circuit-behavior measures are response biomarkers of TMS response and predictive markers of functional/suicidality outcomes, 4) machine-learning methods to discover how our data form naturally organized clusters of TMS response trajectories. We will use principal components analysis for data reduction, clustering algorithms (e.g., hidden Markov models) to identify cohesive subgroups defined by circuit dysfunctions, and GLMs to assess if clusters are differentiated by behavior-symptom-functional-suicidality profiles and TMS-related clinical outcomes and, 5) a whole brain voxel-wise approach to quantify circuits and regions within circuits that might be missed by using a priori circuits and regions of interest.

## Data management

### Behavioral and cognitive data management

Data, once acquired, will be coded and given a generic indicator (e.g. 001). Individuals who are listed on the protocol will have access to all coded study data. Coded data will be shared with participating sites for data analysis. All research staff will undergo training from the lead investigator at each site, including the means through which confidentiality is maintained, the proper procedures as dictated by study protocol, and a review of any operating procedures that are important for data collection and veteran safety and security. Standard operating procedures will be shared with participating sites and an overall training log will be kept up to date to ensure collaborating sites are collecting data and running veteran visits in a standardized way. All information regarding prescription of the treatment parameters are monitored throughout the course of treatment and captured in the VA National Clinical TMS Program Quality Improvement Project. VA HIPAA authorization approved by IRB and embedded in the consent form allows for access to this clinical data.

Shared, coded data will be transferred through a secure file transfer software. The sharing of any PHI, if necessary, over the course of the study, will follow the coordinating sites guidance for best practice. The sharing of VA PHI will happen as permitted by VA HIPAA authorization embedded in VA consent.

### MRI data management

MRI acquisitions will be transferred from each facility to the central facility at Stanford through secure transport. All MRI data will be anonymized including removing sensitive subject information and defacing structural images. The data storage system can only be accessed securely by certain investigators using two factor authentication. The storage system is scalable to large datasets and snapshots are recorded over time to prevent any possibility of data loss.

### Data monitoring and safety reporting

A Data Safety and Monitoring Plan will be in place, consistent with standard protocols at the participating sites. Veteran recruitment, protocol compliance, and adverse events (AEs) will be tracked for each site on a semi-annual basis to monitor veteran safety, study progress, and efficacy; and to make recommendations for study continuation. All AEs will be recorded on standard forms and will indicate the severity, date of onset, and likelihood that the AE is related to a study procedure. The PI will ensure that all measures necessary to resolve the SAE are taken and that the Institutional Review Board is notified as soon as is practical in accordance with local institutional policy.

## Discussion

Despite the wide scale adoption of repetitive transcranial magnetic stimulation, we still lack mechanistically-driven biomarkers designed to identify who is most likely to respond, and why. The identification of more precise solutions for MDD patients is imperative given that pharmacoresistant depression can be life threatening. Our study addresses this need through a systematic evaluation of brain circuit biomarkers in patients taking part in the VA Clinical TMS Program. We use a prospective design to evaluate cognitive control network connectivity as a predictive biomarker of the clinical effect of repetitive transcranial magnetic stimulation, and as a response biomarker of change with TMS.

### Strengths

Innovations in our study design include 1) adequate power to interrogate imaging markers, 2) standardization to minimize variability, 3) implementation of a longitudinal design to quantify TMS-related changes in imaging markers, 4) integration of task-evoked and resting state imaging markers, and 5) establishing the foundations for expanding lessons learned to additional diagnoses and parameters.

#### Adequate Power

Our study will be the first pragmatic, large scale mechanistic trial to use functional connectivity neuroimaging and behavioral biomarkers of cognitive control as targets for response and prediction of outcomes in pharmacoresistant patients. Reflective of the emergence of TMS research, previous neuroimaging studies of TMS have employed small samples (mean n= ~ 24) [10]. Our target of 100 patients will ensure statistical power to test our hypotheses.

#### Standardization to minimize variability

Our study will rigorously standardize TMS and neuroimaging protocols and the analytic pipeline (including stringent motion correction). Drawing conclusions about the utility of neuroimaging biomarkers has been difficult from current knowledge given the juxtaposition of small sample sizes and variability in neuroimaging/connectivity analysis, methods, and TMS delivery. By implementing a standardized approach in a well-powered sample, we will be in a unique position to parse variance due to biomarkers of interest versus variance due to other factors.

#### Implementation of longitudinal design

We will be obtaining fMRI scans at three time points during the course of TMS (baseline, after 1 Week and Post-treatment), while other TMS neuroimaging studies have obtained images at only one (Baseline) or two time points (Baseline and Post-treatment). Our approach will allow us, for the first time, to investigate whether change in functional connectivity of particular neural circuits in response to TMS may serve as an early biomarker (i.e., after only a few TMS sessions) of the subsequent effect of TMS. This information could enable clinicians to discontinue an intensive therapy for certain patients early in the TMS course, allowing the right treatment to be identified more quickly, aborting unnecessary side effects, and lowering the risk of patients disengaging from care due to frustration.

#### Task-evoked and resting state imaging markers

The majority of TMS neuroimaging studies have relied heavily on resting state imaging [10]. Our cognitive control measures will allow us to probe our biomarkers of the DLPFC-anchored cognitive control network, elicited during GoNoGo and working memory tasks, and its connectivity with resting circuits involved in regulation. Additionally, if our broad hypothesis is correct that connectivity and behavioral performance changes are correlated, then behavioral measures may be used as proxies for neuroimaging data in clinical practice. Such a finding would offer a scalable TMS response biomarker that complements our mechanistic understanding based on neuroimaging measures of circuit connectivity.

#### Expanding lessons to additional diagnoses and parameters

Given dysfunction in cognitive control and associated circuits are transdiagnostic [75], our findings will be a foundation for expanding to other psychiatric disorders in future trials. Our proposed sample will be sufficiently representative of the comorbidities in pharmacoresistant MDD patients to facilitate a future such transdiagnostic approach.

### Limitations

Our study design also presents certain limitations including 1) the lack of a control group inherent in the observational design, 2) the definition of biomarkers, 3) the co-administration of neuromodulation and psychotropic medications, 4) presence of cognitive control dysfunction in our study population, 5) use of the Beam-F3 method for stimulation targeting vs. neuronavigation methods, 6) choice of stimulation site and parameters, and 7) the overrepresentation of older, male veterans in our population.

#### Lack of control group

Our study is observational and therefore lacks a control group. Veterans receive the standard TMS protocol for MDD as part of their clinical care through the VA’s Clinical TMS Program.

#### Definition of biomarkers

We follow the BEST (Biomarkers, EndpointS, and other Tools) resource of the FDA-NIH Biomarker Working Group for defining biomarkers [76]. Although we do not have a means to randomize to a treatment control in the current pragmatic design, our stratification in the analysis of veterans based on extent of cognitive control meets the broad definition that a predictive biomarker identifies individuals who are more likely than similar individuals without the biomarker to experience a favorable or unfavorable effect from TMS.

#### Co-Administration of Neuromodulation and Psychotropic Medication

The combination of neuromodulation and medications used for pharmacoresistant major depression adds a degree of complexity to the current trial. We considered recruitment of medication-free veterans, but requiring veterans to be medication-free would not be feasible or ethical. Combined TMS and medications have been shown to be safe and efficacious in veteran patients [77]. Thus, following prior TMS trials, clinical interventions will be stable for at least 6 weeks prior to TMS and during the study. Based on our prior experience, we anticipate medication changes during TMS will be limited. Should medication changes occur during the TMS treatment course, they will be recorded and post-hoc explorations will be performed to evaluate the effect on outcomes.

#### Presence of cognitive control dysfunction in our study population

Patients with pharmacoresistant depression may be the very individuals who demonstrate impaired cognitive control function. Consistent with a dimensional approach, we will undertake analyses based on the continuous degree of cognitive control dysfunction as well as seek to binarize the sample in subsequent analyses.

#### Use of the Beam-F3 method for stimulation targeting

We considered several approaches to target the DLPFC and elected to use individual scalp landmarks to determine the site of stimulation (i.e., Beam-F3 Method [72]). This approach is recommended by the National Network of Depression Centers [78] when frameless stereotaxy is unavailable or impractical. It offers significant advantages over the standard “5-cm rule” that often misses the DLPFC [79]. We recognize that this method has limitations in and of itself, particularly if our goal was discovery of novel approaches to precision targeting. However, recent work indicates the Beam-F3 method provides a reasonable approximation compared to neuronavigation [80]; thus, we consider it suitable for our purposes.

#### Choice of stimulation site and parameters

The pragmatic design of the proposed trial, the need for standardization, and the opportunity to leverage the large-scale Clinical Program necessitate a focus on reproducible parameters, namely 10 Hz DLPFC TMS, which has been the standard clinical protocol for MDD for nearly 10 years. Nonetheless, we recognize that the field is developing quickly. Thus, we anticipate planning thoroughly for future protocol expansions that would include consideration of alternative stimulation parameters, such as lower frequency, theta burst, or accelerated TMS. Our data on systematically evaluated patients will provide an important foundation from which to explore and compare new parameters.

#### Overrepresentation of older, male veterans

Reflecting the veteran population, we anticipate a preponderance of older male participants; however, our recruitment strategy will be targeted to ensure maximum possible recruitment of female veterans. However, because we are leveraging the VA TMS Clinical Program, our distribution will be reflective of the demographic mix within veterans referred to the participating clinics. Recent US Census data estimates the number of women veterans in the US to be approximately 9.2% of the total veteran population [81]. We anticipate a similar proportion of women recruited for this study. Although veteran participants in TMS trials may on average be older than non-veteran participants, older age has not been found to be a predictor of poorer response to TMS in veteran patients [82]. We will also explore secondary hypotheses that TMS-modulated brain-behavior targets are moderated by sex differences within the anticipated male/female distribution in the veteran population.

## Data Availability

Not applicable.

## Abbreviations

AC-PC: Anterior Commissure-Posterior Commissure
ACS: Advanced Clinical Solutions
AE: Adverse Event
AP: Anterior to Posterior
AUDIT: Alcohol Use Disorder Identification Test
BA46: Brodmann Area 46
BEST: Biomarkers, Endpoints and Other Tools
BOLD: Blood Oxygenation Level Dependent
BVMT-R: Brief Visuospatial Memory Test-Revised
C-SSRS: Columbia Suicide Severity Rating Scale
CVLT-III: California Verbal Learning Test- Third Edition
CPT: Continuous Performance Test
D-KEFS: Delis-Kaplan Executive Function System
DLPFC: Dorsolateral Prefrontal Cortex
DMN: Default Mode Network
DUDIT: Drug Use Disorder Identification Test
DSM-III-R: Diagnostic and Statistical Manual for Mental Disorders, Revised 3rd Edition
DSM-5: Diagnostic and Statistical Manual for Mental Disorders, 5th Edition
FA: Flip Angle
FDA: Food and Drug Administration
FIRMM: Framewise Integrated Real-time MRI Monitoring
fMRI: Functional Magnetic Resonance Imaging
FTT: Finger Tapping Test
GLM: Generalized Linear Model
HIPAA: Health Insurance Portability and Accountability Act
IRB: Institutional Review Board
LEC-5: Life Events Checklist for DSM-5
MDD: Major Depressive Disorder
MINI: The MINI-International Neuropsychiatric Interview
MRI: Magnetic Resonance Imaging
MT: Motor Threshold
NIH: National Institutes of Health
NIMH: National Institutes of Mental Health
PA: Posterior to Anterior
PCL-5: Post Traumatic Stress Disorder Checklist for DSM-5
PI: Principal Investigator
PHI: Protected Health Information
POET: Perception of Emotions Test
PROMO: Prospective Motion Correction
PTSD: Post Traumatic Stress Disorder
REDCap: Research Electronic Data Capture
RMS: Root Mean Square
QC: Quality Control
QIDS: Quick Inventory of Depressive Symptoms
QIDS-SR: Quick Inventory of Depressive Symptoms- Self-Report Version
rTMS/TMS: Repetitive Transcranial Magnetic Stimulation
SAE: Serious Adverse Event
SAT: Shifting Attention Test
SDC: Symbol Digit Coding
sgACC: Subgenual Anterior Cingulate Cortex
SCQ: Self-Administered Comorbidity Questionnaire
TE: Echo Time
TMS: Transcranial Magnetic Stimulation
TOPF: Test of Premorbid Functioning
TR: Repetition Time
UHP: Ultra High Performance
US: United States
VBM: Verbal Memory
VA: Veteran Administration
VF: Verbal Fluency
VIM: Visual Memory
VR-36: Veterans’ RAND 36-Item Health Survey
WAIS-IV: Wechsler Adult Intelligence Scale-4th edition

## References

[1] Friedrich MJ (2017). Depression is the leading cause of disability around the world. JAMA.

[2] Williams LM, Goldstein-Piekarski AN, Chowdhry N, Grisanzio KA, Haug NA, Samara Z (2016). Developing a clinical translational neuroscience taxonomy for anxiety and mood disorder: protocol for the baseline-follow up research domain criteria anxiety and depression (“RAD”) project. BMC Psychiatry.

[3] Fava M (2003). Diagnosis and definition of treatment-resistant depression. Biol Psychiatry.

[4] Trevino K, McClintock SM, McDonald Fischer N, Vora A, Husain MM (2014). Defining treatment-resistant depression: a comprehensive review of the literature. Ann Clin Psychiatry.

[5] Garcia-Toro M, Medina E, Galan JL, Gonzalez MA, Maurino J (2012). Treatment patterns in major depressive disorder after an inadequate response to first-line antidepressant treatment. BMC Psychiatry.

[6] Bergfeld IO, Mantione M, Figee M, Schuurman PR, Lok A, Denys D (2018). Treatment-resistant depression and suicidality. J Affect Disord.

[7] Williams LM (2017). Defining biotypes for depression and anxiety based on large-scale circuit dysfunction: a theoretical review of the evidence and future directions for clinical translation. Depress Anxiety.

[8] Sheline YI, Price JL, Yan Z, Mintun MA (2010). Resting-state functional MRI in depression unmasks increased connectivity between networks via the dorsal nexus. Proc Natl Acad Sci U S A.

[9] Gyurak A, Patenaude B, Korgaonkar MS, Grieve SM, Williams LM, Etkin A (2016). Frontoparietal activation during response inhibition predicts remission to antidepressants in patients with major depression. Biol Psychiatry.

[10] Philip NS, Barredo J, Aiken E, Carpenter LL (2018). Neuroimaging mechanisms of therapeutic Transcranial magnetic stimulation for major depressive disorder. Biol Psychiatry Cogn Neurosci Neuroimaging.

[11] Cole MW, Repovš G, Anticevic A (2014). The frontoparietal control system: a central role in mental health. Neuroscientist.

[12] Sylvester CM, Corbetta M, Raichle ME, Rodebaugh TL, Schlaggar BL, Sheline YI (2012). Functional network dysfunction in anxiety and anxiety disorders. Trends Neurosci.

[13] Hamilton JP, Farmer M, Fogelman P, Gotlib IH (2015). Depressive rumination, the default-mode network, and the dark matter of clinical neuroscience. Biol Psychiatry.

[14] Goldstein-Piekarski AN, Staveland BR, Ball TM, Yesavage J, Korgaonkar MS, Williams LM (2018). Intrinsic functional connectivity predicts remission on antidepressants: a randomized controlled trial to identify clinically applicable imaging biomarkers. Transl Psychiatry.

[15] Tozzi L, Goldstein-Piekarski AN, Korgaonkar MS, Williams LM (2020). Connectivity of the cognitive control network during response inhibition as a predictive and response biomarker in major depression: evidence from a randomized clinical trial. Biol Psychiatry.

[16] Mayberg HS, Brannan SK, Tekell JL, Silva JA, Mahurin RK, McGinnis S (2000). Regional metabolic effects of fluoxetine in major depression: serial changes and relationship to clinical response. Biol Psychiatry.

[17] Chen AC, Oathes DJ, Chang C, Bradley T, Zhou ZW, Williams LM (2013). Causal interactions between fronto-parietal central executive and default-mode networks in humans. Proc Natl Acad Sci U S A.

[18] Fox MD, Buckner RL, White MP, Greicius MD, Pascual-Leone A (2012). Efficacy of transcranial magnetic stimulation targets for depression is related to intrinsic functional connectivity with the subgenual cingulate. Biol Psychiatry.

[19] Fox MD, Liu H, Pascual-Leone A (2013). Identification of reproducible individualized targets for treatment of depression with TMS based on intrinsic connectivity. Neuroimage.

[20] Baeken C, Marinazzo D, Wu GR, Van Schuerbeek P, De Mey J, Marchetti I (2014). Accelerated HF-rTMS in treatment-resistant unipolar depression: insights from subgenual anterior cingulate functional connectivity. World J Biol Psychiatry.

[21] Liston C, Chen AC, Zebley BD, Drysdale AT, Gordon R, Leuchter B (2014). Default mode network mechanisms of transcranial magnetic stimulation in depression. Biol Psychiatry.

[22] Weigand A, Horn A, Caballero R, Cooke D, Stern AP, Taylor SF (2018). Prospective validation that Subgenual connectivity predicts antidepressant efficacy of Transcranial magnetic stimulation sites. Biol Psychiatry.

[23] Taylor SF, Ho SS, Abagis T, Angstadt M, Maixner DF, Welsh RC (2018). Changes in brain connectivity during a sham-controlled, transcranial magnetic stimulation trial for depression. J Affect Disord.

[24] Mi Z, Biswas K, Fairchild JK, Davis-Karim A, Phibbs CS, Forman SD (2017). Repetitive transcranial magnetic stimulation (rTMS) for treatment-resistant major depression (TRMD) Veteran patients: study protocol for a randomized controlled trial. Trials.

[25] Yesavage JA, Fairchild JK, Mi Z, Biswas K, Davis-Karim A, Phibbs CS (2018). Effect of repetitive Transcranial magnetic stimulation on treatment-resistant major depression in US veterans: a randomized clinical trial. JAMA Psychiatry.

[26] Berlow YA, Zandvakili A, Philip NS (2020). The clinical utility of imaging-defined biotypes of depression and transcranial magnetic stimulation: a decision curve analysis. Brain Stimul.

[27] Bilder RM, Postal KS, Barisa M, Aase DM, Cullum CM, Gillaspy SR (2020). Inter organizational practice committee recommendations/guidance for Teleneuropsychology in response to the COVID-19 pandemic†. Arch Clin Neuropsychol.

[28] Brearly TW, Shura RD, Martindale SL, Lazowski RA, Luxton DD, Shenal BV (2017). Neuropsychological test administration by videoconference: a systematic review and meta-analysis. Neuropsychol Rev.

[29] Marra DE, Hoelzle JB, Davis JJ, Schwartz ES. Initial changes in neuropsychologists clinical practice during the COVID-19 pandemic: a survey study. Clin Neuropsychol. 2020:1–16. 10.1080/13854046.2020.1800098 32723158

[30] Marra DE, Hamlet KM, Bauer RM, Bowers D (2020). Validity of teleneuropsychology for older adults in response to COVID-19: a systematic and critical review. Clin Neuropsychol.

[31] Sheehan DV, Lecrubier Y, Sheehan KH, Amorim P, Janavs J, Weiller E (1998). The Mini-International Neuropsychiatric Interview (M.I.N.I.): the development and validation of a structured diagnostic psychiatric interview for DSM-IV and ICD-10. J Clin Psychiatry.

[32] Sangha O, Stucki G, Liang MH, Fossel AH, Katz JN (2003). The self-administered comorbidity questionnaire: a new method to assess comorbidity for clinical and health services research. Arthritis Rheum.

[33] Selzer ML (1971). The Michigan alcoholism screening test: the quest for a new diagnostic instrument. Am J Psychiatry.

[34] Skinner HA (1982). The drug abuse screening test. Addict Behav.

[35] Wortmann JH, Jordan AH, Weathers FW, Resick PA, Dondanville KA, Hall-Clark B (2016). Psychometric analysis of the PTSD Checklist-5 (PCL-5) among treatment-seeking military service members. Psychol Assess.

[36] National Center for PTSD. The Life Events Checklist for DSM-5 (LEC-5). Available from: www.ptsd.va.gov. Accessed 21 Apr 2020.

[37] Wechsler D (2008). Wechsler Adult Intelligence Scale.

[38] Korgaonkar MS, Grieve SM, Etkin A, Koslow SH, Williams LM (2013). Using standardized fMRI protocols to identify patterns of prefrontal circuit dysregulation that are common and specific to cognitive and emotional tasks in major depressive disorder: first wave results from the iSPOT-D study. Neuropsychopharmacology.

[39] Barch DM, Burgess GC, Harms MP, Petersen SE, Schlaggar BL, Corbetta M (2013). Function in the human connectome: task-fMRI and individual differences in behavior. Neuroimage.

[40] Friedman L, Glover GH, Consortium F (2006). Reducing interscanner variability of activation in a multicenter fMRI study: controlling for signal-to-fluctuation-noise-ratio (SFNR) differences. Neuroimage.

[41] Falconer E, Bryant R, Felmingham KL, Kemp AH, Gordon E, Peduto A (2008). The neural networks of inhibitory control in posttraumatic stress disorder. J Psychiatry Neurosci.

[42] Tozzi L, Staveland B, Holt-Gosselin B, Chesnut M, Chang SE, Choi D (2020). The human connectome project for disordered emotional states: protocol and rationale for a research domain criteria study of brain connectivity in young adult anxiety and depression. Neuroimage.

[43] Glover GH, Mueller BA, Turner JA, van Erp TG, Liu TT, Greve DN (2012). Function biomedical informatics research network recommendations for prospective multicenter functional MRI studies. J Magn Reson Imaging.

[44] White N, Roddey C, Shankaranarayanan A, Han E, Rettmann D, Santos J (2010). PROMO: real-time prospective motion correction in MRI using image-based tracking. Magn Reson Med.

[45] Dosenbach NUF, Koller JM, Earl EA, Miranda-Dominguez O, Klein RL, Van AN (2017). Real-time motion analytics during brain MRI improve data quality and reduce costs. Neuroimage.

[46] Strother SC, Anderson J, Hansen LK, Kjems U, Kustra R, Sidtis J (2002). The quantitative evaluation of functional neuroimaging experiments: the NPAIRS data analysis framework. Neuroimage.

[47] Siegel JS, Power JD, Dubis JW, Vogel AC, Church JA, Schlaggar BL (2014). Statistical improvements in functional magnetic resonance imaging analyses produced by censoring high-motion data points. Hum Brain Mapp.

[48] Power JD, Mitra A, Laumann TO, Snyder AZ, Schlaggar BL, Petersen SE (2014). Methods to detect, characterize, and remove motion artifact in resting state fMRI. Neuroimage.

[49] Wellcome Centre for Human Neuroimaging, UCL Queen Square Institute of Neurology. SPM Extensions. Available from: http://www.fil.ion.ucl.ac.uk/spm/ext/# TSDiffAna. Accessed 17 Apr 2020.

[50] Friedman L, Glover GH (2006). Report on a multicenter fMRI quality assurance protocol. J Magn Reson Imaging.

[51] Silverstein SM, Berten S, Olson P, Paul R, Willams LM, Cooper N (2007). Development and validation of a world-wide-web-based neurocognitive assessment battery: WebNeuro. Behav Res Methods.

[52] Watters AJ, Williams LM (2011). Negative biases and risk for depression; integrating self-report and emotion task markers. Depress Anxiety.

[53] Kemp AH, Hatch A, Williams LM (2009). Computerized neuropsychological assessments: pros and cons. CNS Spectr.

[54] Gualtieri CT, Johnson LG (2006). Reliability and validity of a computerized neurocognitive test battery. CNS Vital Signs Arch Clin Neuropsychol.

[55] Cole WR, Arrieux JP, Schwab K, Ivins BJ, Qashu FM, Lewis SC (2013). Test-retest reliability of four computerized neurocognitive assessment tools in an active duty military population. Arch Clin Neuropsychol.

[56] Rush AJ, Trivedi MH, Ibrahim HM, Carmody TJ, Arnow B, Klein DN (2003). The 16-item quick inventory of depressive symptomatology (QIDS), clinician rating (QIDS-C), and self-report (QIDS-SR): a psychometric evaluation in patients with chronic major depression. Biol Psychiatry.

[57] Kazis LE, Miller DR, Clark JA, Skinner KM, Lee A, Ren XS (2004). Improving the response choices on the veterans SF-36 health survey role functioning scales: results from the veterans health study. J Ambul Care Manage.

[58] Ware JE, Sherbourne CD (1992). The MOS 36-item short-form health survey (SF-36). I. Conceptual framework and item selection. Med Care.

[59] Posner K, Brent D, Lucas C (2009). Columbia–suicide severity rating scale (C-SSRS).

[60] Wadsworth HE, Galusha-Glasscock JM, Womack KB, Quiceno M, Weiner MF, Hynan LS (2016). Remote neuropsychological assessment in rural American Indians with and without cognitive impairment. Arch Clin Neuropsychol.

[61] Mathersul D, Palmer DM, Gur RC, Gur RE, Cooper N, Gordon E (2009). Explicit identification and implicit recognition of facial emotions: II. Core domains and relationships with general cognition. J Clin Exp Neuropsychol.

[62] Williams LM, Mathersul D, Palmer DM, Gur RC, Gur RE, Gordon E (2009). Explicit identification and implicit recognition of facial emotions: I. age effects in males and females across 10 decades. J Clin Exp Neuropsychol.

[63] Turvey C, Coleman M, Dennison O, Drude K, Goldenson M, Hirsch P (2013). ATA practice guidelines for video-based online mental health services. Telemed J E Health.

[64] Krupinski EA, Antoniotti N, Bernard J (2013). Utilization of the American telemedicine Association's clinical practice guidelines. Telemed J E Health.

[65] Reitan RM, Wolfson D (1993). The Halstead–Reitan Neuropsychological Test Battery: Theory and Clinical Interpretation. 2nd edition ed.

[66] Heaton RK (2004). Revised comprehensive norms for an expanded halstead-reitan battery: demographically adjusted neuropsychological norms for african american and caucasian adults, professional manual.

[67] Dikmen SS, Heaton RK, Grant I, Temkin NR (1999). Test-retest reliability and practice effects of expanded Halstead-Reitan neuropsychological test battery. J Int Neuropsychol Soc.

[68] Ricker JH, Axelrod BN (1994). Analysis of an Oral paradigm for the trail making test. Assessment.

[69] Delis D, Kaplan E, Kramer J (2001). Delis-Kaplan executive function system: Examiner's manual.

[70] Delis D, Kramer J, Kaplan E, Ober B (2017). California verbal learning test.

[71] Benedict R (1997). Brief Visuospatial memory test - revised: professional manual.

[72] Beam W, Borckardt JJ, Reeves ST, George MS (2009). An efficient and accurate new method for locating the F3 position for prefrontal TMS applications. Brain Stimul.

[73] Goldstein-Piekarski AN, Korgaonkar MS, Green E, Suppes T, Schatzberg AF, Hastie T (2016). Human amygdala engagement moderated by early life stress exposure is a biobehavioral target for predicting recovery on antidepressants. Proc Natl Acad Sci U S A.

[74] Philip NS, Barredo J, Tyrka AR, Price LH, Carpenter LL (2018). Network mechanisms of clinical response to transcranial magnetic stimulation in posttraumatic stress disorder and major depressive disorder. Biol Psychiatry.

[75] Forster S, Nunez Elizalde AO, Castle E, Bishop SJ (2015). Unraveling the anxious mind: anxiety, worry, and frontal engagement in sustained attention versus off-task processing. Cereb Cortex.

[76] FDA-NIH Biomarker Working Group. BEST (Biomarkers, EndpointS, and other Tools) Resource. Available from: https://www.ncbi.nlm.nih.gov/books/NBK326791/?report= reader. Accessed 21 Apr 2020. 27010052

[77] Carpenter LL, Conelea C, Tyrka AR, Welch ES, Greenberg BD, Price LH (2018). 5 Hz repetitive transcranial magnetic stimulation for posttraumatic stress disorder comorbid with major depressive disorder. J Affect Disord.

[78] McClintock SM, Reti IM, Carpenter LL, McDonald WM, Dubin M, Taylor SF (2018). Consensus Recommendations for the Clinical Application of Repetitive Transcranial Magnetic Stimulation (rTMS) in the Treatment of Depression. J Clin Psychiatry.

[79] Trapp NT, Bruss J, King Johnson M, Uitermarkt BD, Garrett L, Heinzerling A (2020). Reliability of targeting methods in TMS for depression: Beam F3 vs. 5.5 cm. Brain Stimul.

[80] Mir-Moghtadaei A, Caballero R, Fried P, Fox MD, Lee K, Giacobbe P (2015). Concordance between BeamF3 and MRI-neuronavigated target sites for repetitive Transcranial magnetic stimulation of the left dorsolateral prefrontal cortex. Brain Stimul.

[81] United States Census Bureau, American Community Survey 2018. Veteran Status. Available from: https://data.census.gov/cedsci/all?q=veterans&hide Preview=false&tid =ACSDT1Y2018. B21001&t=Veterans&vintage=2018. Accessed 21 Apr 2020.

[82] Conelea CA, Philip NS, Yip AG, Barnes JL, Niedzwiecki MJ, Greenberg BD (2017). Transcranial magnetic stimulation for treatment-resistant depression: naturalistic treatment outcomes for younger versus older patients. J Affect Disord.

